# Intermittent and Periodic Fasting, Hormones, and Cancer Prevention

**DOI:** 10.3390/cancers13184587

**Published:** 2021-09-13

**Authors:** Giulia Salvadori, Mario Giuseppe Mirisola, Valter D. Longo

**Affiliations:** 1 Department of Oncology and Hemato-Oncology, University of Milan, 20122 Milan, Italy; giulia.salvadori@ifom.eu; 2 IFOM, FIRC Institute of Molecular Oncology, 20139 Milan, Italy; 3 Department of Surgical, Oncological, and Oral Sciences, University of Palermo, 90127 Palermo, Italy; mario.mirisola@unipa.it; 4 Department of Biological Sciences, Longevity Institute, Leonard Davis School of Gerontology, University of Southern California, Los Angeles, CA 90089, USA

**Keywords:** fasting, growth hormones, aging, DNA damage, cancer prevention

## Abstract

**Simple Summary:**

Hormonal and growth factor alterations, related to an elevated food consumption and excessive adiposity, affect the regulation of genes involved in cellular processes including proliferation, differentiation and DNA repair, allowing cells to survive and proliferate despite the accumulation of mutations which lead to malignant transformation. The growth hormone/insulin growth factor-1 (GH/IGF-1)/ insulin pathway and its downstream effectors, in fact, are known to promote aging and/or age-related diseases, including cancer, in many model organisms. The restriction of nutrients is established to have strong effects on levels of hormones and growth factors, delaying the incidence of age-related diseases and prolonging lifespan. Here, we summarize the effects caused by different nutrition intervention strategies on cellular damage, aging and cancer.

**Abstract:**

The restriction of proteins, amino acids or sugars can have profound effects on the levels of hormones and factors including growth hormone, IGF-1 and insulin. In turn, these can regulate intracellular signaling pathways as well as cellular damage and aging, but also multisystem regeneration. Both intermittent (IF) and periodic fasting (PF) have been shown to have both acute and long-term effects on these hormones. Here, we review the effects of nutrients and fasting on hormones and genes established to affect aging and cancer. We describe the link between dietary interventions and genetic pathways affecting the levels of these hormones and focus on the mechanisms responsible for the cancer preventive effects. We propose that IF and PF can reduce tumor incidence both by delaying aging and preventing DNA damage and immunosenescence and also by killing damaged, pre-cancerous and cancer cells.

## 1. Introduction

Many dietary patterns, including the Western diet, are associated with reduced lifespan and health span and appear to affect cancer incidence by two major hormonal axes/pathways: (1) the growth hormone-IGF-1; (2) the insulin signaling [1,2,3,4,5,6,7].

Higher protein intake increases the release of growth hormone releasing hormone, and consequently growth hormone release from the pituitary gland and IGF-1 release primarily from the liver [8]. High IGF-1 has been associated with elevated incidence of a number of cancers [8,9,10,11]. On the other hand, excessive carbohydrate and/or fat intake can result in excess adiposity, which is associated with high oxidative stress, inflammation, alterations in hormones and growth factors’ production, acquisition of insulin resistance and consequently hyperinsulinemia [12,13,14]. Overweight women are reported to frequently present insulin resistance and low plasma levels of sex hormone-binding globulin which lead, as a consequence, to an increase in total and free sex hormone levels [15]. Hyperinsulinemia, in fact, blocks the production of sex hormone-binding globulin by the liver and, moreover, is associated with an increased production of androgens which are reported, together with estrogens, to stimulate the development and growth of several cancers [16]. Furthermore, insulin sustains insulin like growth factor 1 (IGF-1) activity, partly through the reduction of IGF binding protein 1 (IGFBP-1) synthesis, and elevated GH-IGF-1 increases insulin levels and resistance [5,6,17]. Not surprisingly, both elevated concentrations of insulin and IGF-1 are associated with multiple cancer types, including breast, endometrium, pancreas and colon [3,4,7,18]. However, it is not clear whether insulin and IGF-1 may promote cancer directly by promoting growth and preventing apoptosis, or by accelerating the aging process, which is a key risk factor for many cancers.

Nutrient sensing pathways regulate metabolism as well as growth and promote aging, aging-related diseases and genomic instability in several organisms, ranging from the simplest eukaryotes to mammals [19]. Studies in these model organisms show that impairment of genes that promote growth can extend lifespan, suggesting that age-related diseases, including cancer, could be postponed or prevented by switching from a pro-growth mode to a maintenance mode [20,21]. The GH/IGF-1 pathway and its downstream effectors including target of rapamycin (TOR), protein kinase A (PKA) and the ribosomal protein S6 kinase (S6K) are reported to promote aging and age-related diseases, including cancer, in several model organisms [22]. 

Thus, the understanding of nutrient-response pathways can provide insights on the mechanisms linking food intake to age-related diseases. 

### 1.1. Growth Genes, Longevity, and Cancer: From Yeast to Humans

#### 1.1.1. Growth Genes Aging and DNA Damage in Yeast

Yeast has been successfully used as a model system to understand the molecular basis of some of the most basic and important cellular processes. It is therefore not surprising that the aging research field included this simple unicellular organism among the most important organisms for identifying and studying the central pathways regulating longevity. Its short lifespan, low experimental costs and, more importantly, the availability of powerful and rigorous genetic methodologies and high throughput technologies, render it ideal for molecular genetics of aging studies. In addition, even considering the great evolutionary distance between yeast and mammals, comparative genomics highlighted that about 30% of the human genes involved in diseases have a yeast orthologue [23]. 

In 1959 Mortimer and Johnston demonstrated that a single yeast cell can divide a limited number of times [24]. This discovery was eventually used to screen and identify genomic mutations capable of affecting the replicative potential of a single yeast cell (replicative lifespan, RLS) [25].

More recently, the ability of post-diauxic yeast cells to survive and form new colonies has been successfully used to measure the yeast chronological lifespan (CLS) [19,26]. Thus, RLS is the yeast counterpart of the Hayflick limit observed in mammalian cell cultures, whose measurement reveals the doubling ability of cultured cells, while the CLS is more closely related to the chronological lifespan of higher eukaryotes. It is worthwhile to note that, even though the two methods could identify different processes, some of the pathways identified, including the Tor and serine-threonine Sch9 kinase (Tor-S6K) and the Ras-adenylate cyclase (AC) and PKA pathway (Ras/AC/PKA), have similar effects on the extension of the two lifespan methods [24,25,27,28]. Studies performed in the yeast *Saccharomyces cerevisiae* showed that deletion of the gene coding the small G protein Ras2 increases resistance to multiple stresses and doubles chronological lifespan [29]. Many data point to the PKA pathway as the Ras-dependent central regulator of longevity in yeast. In fact, mutations impairing adenylate cyclase (AC), a central effector of PKA pathway, also increase yeast longevity. In addition, the key stress resistance transcription factors Msn2/4 are inhibited by PKA activity and Msn2/4 impairment abolishes the pro-longevity effect of Ras2 deletion confirming thus PKA as central regulator of longevity [30].

Genome wide screen of deletion mutants obtained by transposon mutagenesis discovered Sch9-Tor pathway as perhaps the most potent pro-aging pathway in yeast [20]. *SCH9*, which was originally isolated as a suppressor of impaired Ras pathway [31], codes a serine-threonine protein kinase ortholog of mammalian S6K whose deletion increases resistance to multiple stresses and lifespan. The partial overlap with the PKA pathway is further confirmed by the phenotype reversion observed in *sch9* deletion mutant after PKA hyperactivation. However, the overlap between these two pathways is not complete since the contemporary deletions of *RAS2* and *SCH9* has a greater effect on stress resistance as well as viability with respect to each single deletion. More comprehensive genome wide analysis performed using each of the aforementioned aging paradigms identified other genes whose deletion positively affected longevity. Deletion of genes involved in protein synthesis, such as protein component of ribosomal subunits, or genes involved in nuclear export of tRNA identified the transcription factor Gcn4 as another longevity regulator [32]. It must also be noted that these effects are not additive to Tor-Sch9 suggesting common aging regulatory pathways. Deletion of the tRNA wobbling regulator *TRM9* was also identified as a lifespan regulator but its role was argued to be dependent on lower translation efficiency [33].

It is interesting to note that both Ras/AC/PKA and Tor/Sch9/S6k pathways are activated by nutrients and their depletion results in inhibition of these signal transduction pathways [34]. In yeast, the two macronutrients glucose and amino acids activate Ras and Tor, respectively. Reduced glucose supplementation in the growth media is capable of increasing stress resistance, reducing genomic mutation rate and increasing lifespan in a Ras-dependent manner. On the other hand, the restriction of certain amino acids is capable of increasing lifespan by reducing Tor-Sch9/S6k signaling. Interestingly only specific amino acids (methionine, serine, threonine and valine) can activate the Tor-Sch9 pathway and their restriction (each one of them alone or combined) is capable of increasing lifespan as well as stress resistance and decreasing DNA damage [34].

Moreover, the activation of transcription factors Msn2 and Msn4 in *S. cerevisiae* deficient in the Ras/cAMP/PKA signaling makes cells more resistant to stress, in part by inducing the expression of genes encoding for several heat shock proteins, catalase (Ctt1), and the DNA damage inducible gene *DDR2* [20,35]. These results suggest that the effect of mutations in the Tor-Sch9/S6k and Ras/AC/PKA pathways is partly mediated by the regulation of antioxidant defenses and the reduction of oxidants. In fact, yeasts expressing constitutively active *RAS2* oncogene present a lower resistance to oxidants and a decreased lifespan [36].

#### 1.1.2. Growth Genes Aging and DNA Damage in Worms and Flies

The role of nutrient-response pathways was also examined in the worm *Caenorabditis elegans* and fruit fly *Drosophila melanogaster*. Studies of the nematode *Caenorabditis elegans* showed that a reduction of the insulin/insulin-like growth factor signaling pathway (IIS) and the consequent activation of the Forkhead FoxO transcription factor daf-16 which, similarly to Msn2/4 in yeast, regulates genes involved in the cellular stress response and detoxification of xenobiotics and free radicals, extends longevity [37,38,39]. The extension of lifespan in worms also requires the heat shock factor hsf-1, which regulates the expression of heat shock proteins [40]. As observed in yeast, inhibition of TOR-S6 kinase signaling can increase lifespan in worms; in particular, TOR pathway inhibition can activate the process of autophagy and alter the activity of other TOR targets, such as the hypoxia-inducible factor 1 (HIF-1) transcription factor, also independently shown to extend lifespan [39,41].

Moreover, in *C. elegans* mutated in gld-1, a female germline-specific tumor suppressor gene, germ cells can proliferate uncontrolled and form tumors [42]. Mutations in the IGF-1-receptor-like daf-2 gene can protect gld-1 mutants against tumor formations, by reducing germ cell proliferation and inducing cell death through a mechanism which requires daf-16 and cep-1, the orthologue of the mammalian p53 genes [42]. Furthermore, in worms, a mutation in the age-1 PI3K gene, downstream of the daf-2 receptor, was shown to increase mean lifespan by 65% and maximum lifespan at 25 degrees by 110%, suggesting that daf-2 and age-1 act in the same pathway to reduce thermotolerance and antioxidant defenses apparently through a mechanism which involves the stress resistance transcription factor daf-16 [37,43]. Similarly to *ras2*, *cyr1* and *sch9* yeast mutants, which reduce Sod2 expression and require it to extend lifespan, *C. elegans* daf-2 is reported to decrease oxidative stress resistance, in part by down-regulating mitochondrial Mn-SOD gene expression and several heat shock proteins [44]. 

Many studies suggest that orthologs of the yeast and worm genes regulate fruit flies’ longevity as well. *Drosophila melanogaster* impairment of the insulin receptor, in fact, controls the germline stem cell division and cysts growth by a cell autonomous mechanism [45]. The fly daf-16 orthologue FOXO, which is involved in lifespan extension, blocks phosphatidylinositol-3 kinase (PI3K) effect on cell number and the down-regulation of IGF-1 or insulin-AKT/PKB axis, which is reported to promote cancer growth and metastasis in fruit flies, can extend lifespan by up to 85% and inhibit uncontrolled cell growth [46,47,48]. As in *S. cerevisiae* and *C. elegans*, in *D. melanogaster* the inhibition of TOR pathway activity, genetically or by rapamycin treatment, increases lifespan partly by activating autophagy, reducing S6K activity and increasing stress resistance [49]. Analogously to yeast and worms, fruit flies with mutations in the insulin/IGF-1 pathway increase SOD expression and increased survival [47,50]. Furthermore, mutation in the G-protein-coupled receptor homolog MTH gene results in a 35% increase in lifespan and resistance to starvation and superoxide toxicity in *Drosophila melanogaster* [51].

Thus, in yeast, worms and flies, the activation of nutrient signaling pathways is tightly linked to oxidative stress, DNA damage and either increased growth or tumors.

#### 1.1.3. Growth Genes, Aging and Cancer in Mice

Studies in simple model organisms were fundamental to identify conserved nutrient-response pathways that regulate longevity but mouse research on genetics of aging and cancer had also been proceeding in parallel with the studies in simple organisms and reached similar conclusions. Mice studies substantially confirmed the observations made in yeast and other simple aging model systems that certain pro-growth signal transduction pathways activated by nutrients had central roles in aging and cancer. Mutations or deletion in GH or IIS genes, as well as S6K1, can also extend lifespan in mice and reduce age-related diseases including insulin resistance and immune and motor disorders [22,52]. Moreover, a decrease in the PKA signaling pathway in mice extends longevity and reduces the incidence of age-dependent tumors [53]. Mice with homozygous mutations in Prop-1 or Pit1 genes present a deficit in normal pituitary development, which causes a decrease in GH, prolactin and thyroid stimulating hormone (TSH) production; as a consequence, these mice are much smaller than wild-type controls but live ~40% longer [54]. Dwarf mice with a 90% reduction in IGF-1 level or mice carrying heterozygous mutation in IGF-1 receptor (IGF-1R) also present a ~33% increase in life expectancy compared to control mice [55,56]. Several reports provided evidence that a decline in insulin or IGF-1 levels reduces the incidence of spontaneous tumors in mice, thus confirming the central role of IGF-1 as growth promoter and tumorigenesis driver [9]. Transgenic mice expressing IGF-1 are reported to present higher incidences of tumors in mammary glands while transgenic mice overexpressing human GH exhibit hepatic upregulation of GH-signaling mediators which lead to liver neoplasms [57]. Similarly, transgenic mice expressing a constitutively active form of IGF-1R showed aberrant development of the mammary glands and early development of salivary and mammary adenocarcinomas [58]. Conversely, growth hormone receptor binding protein (GHR/BP) knock out mice displayed a lower incidence and a delayed occurrence of neoplastic lesions compared to wild-type littermates, in particular lymphomas and pulmonary carcinomas [59]. Moreover, transgenic animals expressing the GH antagonist G120 GH had lower IGF-1 levels and exhibited decreased tumor incidence in the mammary gland relative to control mice after being treated with the carcinogen 7,12-dimethylbenz(a)anthracene (DMBA) [60]. Accordingly, mice bearing MCF-7 xenografts treated with pegvisomant, which is the clinical version of the G120R GH antagonist able to completely inhibit both GH and IGF-1 signaling in the mammary gland, displayed a 70–80% decreased of circulating IGF-1 and a 30% decrease in tumor size [61]. This effect may be partly due to an increased resistance to oxidative damages and a higher expression of antioxidant enzymes, which are associated with a decrease in IGF-1 levels [19]. Normal levels of GH and IGF-1 could also promote cancer by increasing spontaneous genomic instability through a RAS or AKT hyperactivation dependent mechanism. PTEN inactivation in the mouse prostate causes AKT constitutive activation and is associated with 70% of primary prostate cancers. Moreover, AKT hyperactivation by oncogenic mutations can alter also p53 expression by causing growth-independent hyper-replication and an increase in DNA damage [62]. 

**Figure 1:**
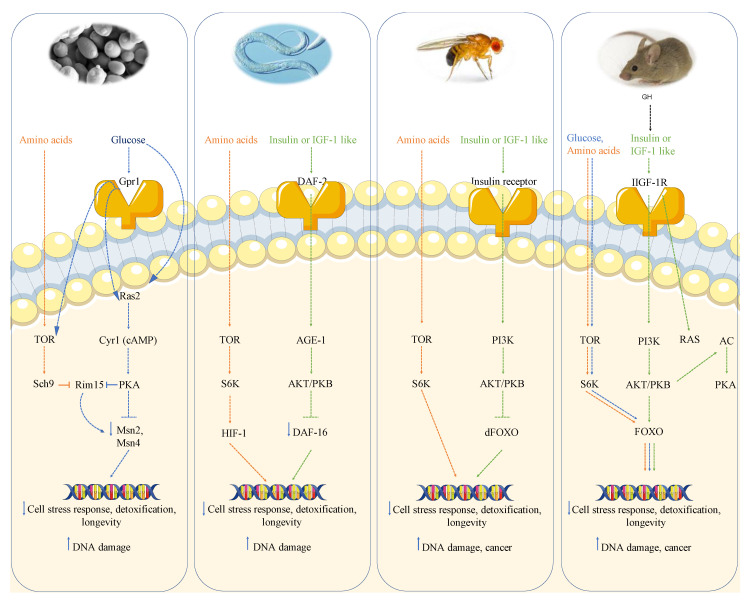
Conserved nutrient signaling pathways in yeast, worms, flies and mammals. A schematic model of the conserved nutrient-signaling pathways that regulate stress response mechanisms, DNA damage, longevity and cancer in different model organisms [22]. In *Saccharomyces cerevisiae*, glucose and amino acids activate Ras/AC/PKA and TOR/Sch9 pathways, respectively. Their activation leads to serine-threonine kinase Rim15 inhibition and consequently to a lowering in Msn2/Msn4 stress resistance transcription factors. These mechanisms promote aging in part by decreasing cell stress response and repair thus increasing DNA damage [20]. In *Caenorhabditis elegans*, insulin/IGF-1 receptor like (daf-2) signaling, through the activation of AKT/PKB pathway, inactivates the Forkhead FoxO transcription factor daf-16, which is involved in the regulation of genes implicated in the cellular stress response and protection against free radicals. As in yeast, also in worms, amino acids can activate the TOR/S6K pathway, accelerating the aging process [44]. Analogously to worms, in Drosophila melanogaster growth factors and amino acids activate AKT/PKB and TOR/S6K pathways, respectively [47]. The activation of TOR/S6K, PI3K/AKT and Ras/AC/PKA pathways, mediated by glucose, amino acids and IGF-1 like signaling, is also maintained in rodents and other mammals, suggesting that these nutrient-sensing pathways, involved in longevity and stress-response mechanisms, are conserved, in part, from the simplest model organism to humans [22,63].

In summary, in mice there is very strong evidence for the link between high growth hormone and IGF-1 levels, DNA damage and cancer, likely mediated at least in part by the activation of AKT, TOR-S6K and PKA signaling, analogously to what is observed in yeast (Figure 1).

#### 1.1.4. Growth Genes and Cancer in Humans

Alterations in GH-IGF-1 axis have also been studied also in humans. Notably, human cancers are frequently mutated in the IGF-1R (2.48% of all cancers) and in its downstream signaling proteins Ras (19% of all cancers) and Akt (1.8% of all cancers) [64,65,66,67]. In agreement with mouse studies, the modulation of the GH-IGF-1 pathway appears to have a key role in cancer prevention in humans. High levels of IGF-1 are, in fact, associated with an increased incidence of several cancers, including colorectal, prostate and breast cancers, while mutations in the human IGF-1R were found to protect against age-related disorders [9,68]. Recent evidence reports that centenarians most frequently present heterozygous mutations in the IGF-1R gene, associated with low IGF-1 serum levels and a higher IGF-1R activity compared to controls characterized by high IGF-1 serum levels [69]. The role of GH/IGF-1 axis activity on longevity and aging-related diseases in human is also supported by long-term studies of an Ecuadorian cohort affected by Laron syndrome (LS) which is characterized by GHR deficiency and consequently is responsible for a 90% reduction of the IGF-1 levels. Guevara-Aguirre et al., monitoring LS patients for more than 20 years, reported that the relation between pro-growth signaling pathways, oxidative stress, genomic instability and cellular damages shown in several model organisms is also observed in humans and human cells [6]. The results from the Ecuadorian cohort of LS patients showed that these individuals are protected from age-related pathologies, in particular cancer and type 2 diabetes, similarly to the results obtained from GH-deficient mice, which are characterized by a 49% decrease in neoplasms’ incidence and an increase in insulin sensitivity compared to control mice [6,59,70,71,72,73,74]. Analysis performed on human mammary epithelial cells (HMECs) incubated in medium supplemented with 15% of LS patients’ serum showed that these cells are characterized by reduced levels of RAS, PKA and TOR, leading to reduced DNA damage and increase apoptosis upon stimulation with oxidative stress compared to cells incubated with 15% of serum derived from LS patients’ relatives, not affected by GHR deficiency [6]. These data suggest that reduced GH-IGF-1/insulin signaling protects from cancer in part by reducing DNA damage and in part by increasing apoptosis in damaged cells, making the link between decreased activity of growth signaling pathways and DNA protection or repair mechanisms conserved from yeast to humans. 

### 1.2. Calorie Restriction (CR) and Cancer

Calorie restriction (CR), a dietary intervention that reduces calorie intake without inducing malnutrition, is the most studied intervention able to extend lifespan but also well established to postpone or even prevent age-related diseases, including cancer [75] (Table 1). Several studies showed that CR increases lifespan in multiple organisms including yeast, flies, worms, rodents and monkeys, protecting from disorders and decline in functions related to aging [22,30,76,77,78,79,80,81]. Curiously, the first study on calorie restriction was conducted in mammals and not in a simple model system. McCay et al. presented for the first time the effects of CR in retarding aging, by increasing lifespan by ~35%, reducing the incidence of kidney disorders, chronic pneumonia and tumors [82] in mice. Similar results were obtained in several studies performed in different rodent models and the underline mechanisms could be mediated by the decrease in blood glucose, IGF-1 and insulin levels, the increase in glucocorticoids and insulin sensitivity as a result of the homeostatic response to reduced body fat stores and an increase in gluconeogenesis [77,83]. Long-term CR is reported to reduce IGF-1 serum levels in rodents by ~30–40%, protecting them against several types of cancers, while treatments with GH or IGF-1 can reverse this protective effect mediated by CR, confirming the important role of these factors in cancer pathogenesis [84,85,86,87]. The NF-E2-related factor 2 (Nrf2) transcription factor has been shown to mediate, in part, the CR anticancer effects. Nrf2, in fact, when activated, improves the activity of several antioxidant and carcinogen-detoxification enzymes, and the anticancer effects of CR is highly impaired in mice deficient in Nrf2 when exposed to carcinogens [88]. These results further demonstrate the effect of CR in increasing resistance to oxidants and other toxins. 

In addition, CR, as well as reduced levels of IGF-1, can decrease genomic instability via Ras- or phosphatidylinositol-3 kinase (PI3K)/Akt/Tor/S6K-dependent mechanisms, which contribute to reducing cancer incidence [89,90] in agreement with what was shown in yeast [20,30]. 

Studies in different rodent models showed that CR is able to reduce the incidence and delay the onset of spontaneous or chemically induced cancers, while in rhesus monkeys, lifelong CR reduces cancer incidence by 50% [91,92,93]. Many beneficial anticancer effects have been achieved by reducing caloric intake by 10 to 50% in mammals [22]. However, more limited restrictions are also capable of affecting longevity in mice. It was demonstrated that the macronutrients ratio has an important role in longevity and a 30% lifespan increase was observed when the protein to carbohydrate ratio is decreased [94]. In fact, protein intake affects the incidence and growth rate of melanoma and breast tumors, probably by activation of the GHR-IGF-1 signaling described earlier [10].

In addition to continues dietary restrictions, the shortage of food without malnutrition for a period of life could have long-term consequences. At least two events were associated with long-term and severe calorie restrictions in human history: Danes faced a 2-year CR without malnutrition, during the first world war, while Norwegians were forced to CR without malnutrition for 4 years in a row during the Second World War. Even though specific cause mortality is not reported in the first case and only circulatory disease are monitored in the second study, in both cases, CR was associated with a strong reduction of mortality rate, 34% and 30%, respectively, suggesting an effect on cancer incidence as well [95,96]. More recent studies on human spontaneously adhering to CR, report slower metabolism, decreased oxidative damages, enhanced DNA damage repair processes and the reduction of diabetes risk factors, cardiovascular diseases and cancer [97]. Moreover, by reducing body weight, CR improves multiple metabolic and hormonal alterations associated with excessive adiposity, including a decrease in visceral and hepatic fat and a reduction in circulating insulin levels, which, consequently, is associated with an increase in sex hormone binding globulin and free hormones like estrogens and testosterone [13,15,16]. The loss of weight mediated by CR also reduces several markers of oxidative and DNA damages; weight loss in obese man is associated with an increase in telomere length in rectal tissue biopsies, suggesting that CR could contribute to the prevention of telomere shortening [98].

Despite the key role of GH/IGF-1 inhibition in increasing life expectancy and reducing or delaying age-related disorders, mimicking the effect of CR, the two approaches may be acting through mechanisms that are only partially overlapping. CR, in fact, can further extend lifespan in GHR-deficient mice, possibly by reducing side effects such as fat accumulation and disorders related to obesity [19,99]. 

Notably, even though the lipid profile is improved and oxidative DNA damage is significantly decreased in calorie-restricted patients, the levels of important hormones associated with cancer, such as IGF1, were unchanged or only slightly changed, possibly because many CR subjects consume high levels of proteins, which regulate IGF-1 levels [100]. Notably, CR provides both a wide range of beneficial effects, as well as detrimental effects including low weight and loss of lean body mass as well as immunosuppression and potentially increased susceptibility to certain infections [101,102,103,104]. Not surprisingly, lifelong CR either caused no effect or a small effect on lifespan in monkeys in spite of showing strong effects on age-related disease-dependent mortality, suggesting that the beneficial effects of CR may be counterbalanced by its detrimental ones.

### 1.3. Fasting and Fasting Mimicking Diets

Because CR can have both very positive and negative effects and is unlikely to be adopted by a significant portion of the populations since it unavoidably causes severe weight loss, intermittent and periodic fasting are emerging as novel interventions which could maintain many of the beneficial effects of CR while reducing the burden and many of the side effects. Fasting, the complete elimination of nutrients from the diet, is the most extreme of the dietary restrictions.

Fasting methods (Table 2). Fasting can be performed for short-term frequent periods, called intermittent fasting (IF), or less frequent but longer periods, known as prolonged and periodic fasting (PF) [63,105]. There are multiple examples of IF diets, including: complete fasting every other day (also called alternate-day fasting ADF); 70% energy restriction every other day; time-restricted feeding (TRF), during which food intake is restricted to 6–12 h per day; and the 5:2 diet, which is achieved by consuming only 500–700 calories for two days a week [106,107,108,109,110]. Thus, IF interventions usually include a phase during which only water is consumed or calorie intake is extremely low, followed by a normal feeding phase which lasts between 12 and 72 h. PF periods, instead, in most cases refer to 2–5 days of water-only fasting or 4–7 days of a fasting mimicking diet (FMD), a diet designed to mimic the metabolic effects induced by fasting [111,112]. Differently from IF, PF does not need to occur at specific intervals and in most cases can be carried out only for few times per year [113]. All these types of fasting cause different degrees of metabolic changes, including the decrease in blood glucose levels, the reduction of glycogen stores, the decrease in circulating leptin levels and the mobilization of fatty acids accompanied by the generation of ketone bodies [63,113]. Moreover, fasting or FMD periods can lead to behavioral changes, including increased awareness, attention, mental acuity, vigilance and feelings of euphoria, thus lowering depressive symptoms [114,115]. 

Fasting can extend lifespan and protect from age-related disorders, including DNA damage or cancer, in different model organisms [63].

*Escherichia coli* bacteria cultured in a calorie-free broth instead of a nutrient-rich one present a fourfold increase in lifespan, while yeast *Saccharomyces cerevisiae* grown in water instead of medium supplemented with glucose present a decrease in Tor/S6K and Ras/AC/PKA nutrient signaling pathways activity and an increased activation of Msn2/4 stress resistance transcription factors [22,30,116]. In the nematode *Caenorabditis elegans*, food deprivation conditions reached by feeding worms with little or no bacteria increase lifespan through mechanisms which involve AMPK, the stress resistance transcription factor DAF-16 [117,118] and the small GTPase RHEB-1 [119]. In rodents, alterations in metabolic and growth factors turned out to be different in accordance with the different forms of fasting applied and also the age at which cycles of fasting were started [120]. IF cycles applied to middle-aged rats increased lifespan by 30–40% compared to rats subjected to a normal dietary regimen [121]. Moreover, in rodents, IF has been shown to prevent and revert metabolic syndrome aspects, reduce abdominal fat, insulin resistance and protects against renal and liver injuries [52,122,123]. 

However, there have been reports showing also IF adverse effects in rodent models. A preclinical study showed that rats subjected to IF for one month had improved glucose tolerance, while rats subjected to 8 months of IF achieved impaired glucose tolerance [124]. Moreover, IF has been shown to have adverse effect on glucose metabolism in hypercholesterolemic mice, contradictory to the beneficial effects of IF on lipid and glucose metabolism shown in other rodent and human studies [125,126,127]. Another preclinical study showed that IF, introduced at 1.5 or 10 months of age, was not able to reduce body weight in A/J mice, compared to controls, and did not increase mean and maximum lifespan when started at 1.5 months. In addition, the same study showed that IF, when started at 10 months of age, decreases mean and maximum lifespan compared to control mice [128]. These results suggest that IF effects on body weight and lifespan are variable and depend on the genotype and age on initiation.

On the other hand, PF has been shown to improve glucose tolerance and delay age-associated diseases in mice, in particular by potentiating cellular resistance and affecting the GH/IGF-1 axis [63,113,129]. In mice, 24–72 h of fasting cause a 30% and 40% decrease in circulating insulin and glucose, respectively, lower IGF-1 levels by 70% and cause a major increase in IGFBP-1 [130]. Moreover, PF causes the down-regulation of TOR-SK6 and eAC-PKA nutrient signaling pathways, and decreases the PI3K-AKT pathway activity [129,130]. Additionally, in humans, PF can lead to a major decrease in circulating IGF-1 and a 5- fold increase in IGF binding protein 1(IGFBP1) [131]. These effects are largely mediated by protein restriction and particularly to the restriction of essential amino acids: in fact, in humans, chronic caloric restriction is not associated with reduced IGF-1 levels unless combined with protein restriction [22,97,132].

PF and especially FMDs, because of their periodic use, limited burden on human subjects, and effects on IGF-1, insulin, glucose, IGFBP-1 and ketone bodies levels, have the potential for applications in cancer prevention and treatment [10,133,134,135].

### 1.4. Fasting Mimicking Diet, Hormones and Cancer Prevention

Several studies indicate that PF is a much more viable strategy than a continuous CR, for cancer prevention and treatment in humans because: (1) it cause a much more extreme set of metabolic changes than CR, including IGF-1, insulin, leptin and glucose decreases, which can be combined with standard of care drugs to promote strong anticancer effects and cancer-free survival; (2) it stimulates anticancer immune responses; (3) it prevents muscle loss; (4) it is amenable to combination with standard cancer treatments but also cancer preventions since it is only conducted for several days periodically and does not require dietary changes between periodic fasting cycles. Preliminary clinical data report that 48 h of fasting are necessary to obtain relevant clinical effects in oncology, such as preventing DNA damages induced by chemotherapy in healthy tissues and improve quality of life to cancer patients [136,137,138]. However, most patients undergoing water-only fasting during cancer treatment had difficulties with sustaining water fasting and reported side effects such as headache, nausea, light-headedness, anemia and weakness [139]. Thus, water-only fasting and intermittent fasting which are expected to be repeated every other day or twice a week, remain a challenging option for the majority of population, especially frail and older subjects and cancer patients. FMD is a plant-based caloric-restricted alimentary regimen (typically between 300 and 1100 kcal per day) characterized by low proteins and sugars and relatively high unsaturated fats. It was developed to mimic many of the metabolic effects induced by water-only fasting but with reduced nutritional risk and burden [112,138,140,141,142]. 

#### 1.4.1. Mice Studies 

Preclinical studies conducted in rodents show that middle-aged mice subjected bimonthly to 4 days of FMD present a 40% decrease in blood glucose levels and a ~9-fold increase in ketone bodies production, suggesting that this regimen is able to mimic the metabolic switch induced by water fasting [112]. Furthermore, 4 days of FMD lead to a 10-fold decrease in insulin level, reduce IGF-1 level by 45% while increase IGFBP-1 by ~8-fold, similarly to the effects of water fasting. Notably, many of these parameters return to normal levels within 7 days of re-feeding [112] but some, including IGF-1 and leptin, do not, suggesting chronic effects of these periodic interventions [141]. Moreover, bimonthly FMD cycles lasting 4 days, started in middle-aged mice, can extend lifespan and positively affect mice health-span by reducing visceral fat deposits, leading consequently to a reduction in body weight, without affecting lean body mass, and decrease multiple organ weight, possibly promoting their regeneration upon refeeding. Middle-aged mice subjected to FMD cycles twice a month for 4 days display a 45% reduction in neoplasia incidence and a ~50% decrease in inflammation-associated skin lesions occurrence when compared to mice fed ad libitum with standard diet. Notably, the FMD also postponed neoplasm-related death by over 3 months and reduced the number of animals with multiple abnormal lesions, suggesting that the FMD regimen not only prevents neoplasia formation but also delays their onset [112].

Studies carried out on bone-marrow-derived stem and progenitor cells show that FMD can also promote immune system regeneration and rejuvenation thus reducing immune-senescence which could be important for cancer development [112,129]. 

Fasting/FMD, partly by reducing PKA activity, circulating IGF-1 and glucose levels and by regulating genes involved in DNA repair (REV1) and cell death (p53), enhances chemotherapeutic agents’ efficacy against multiple kind of tumors, while inducing the protection of normal cells from their toxic side effects [111,129,130,143]. Fasting-dependent reduction in IGF-1 levels was found to be fundamental to protect primary glia and neurons, but not glioma and neuroblastoma cells, from chemotherapies and pro-oxidative compounds [130]. In addition, fasting/FMD, by promoting the switch of cancer cell metabolism from aerobic glycolysis to oxidative phosphorylation (OXPHOS), increases ROS production, contributing to making cancer cells more sensitive to chemotherapy, while protecting normal cells [143]. The FMD-dependent reduction in blood insulin, IGF-1 and leptin, which consequently leads to the inhibition of the PI3K/AKT, mTOR pathways, can also enhance the efficacy of estrogen therapies against estrogen receptor positive (ER^+^) breast cancer [141]. Furthermore, FMD can reduce the expression of Heme-Oxygenase 1 (HO-1), protein which confers protection against oxidative damages and apoptosis, in *in vivo* xenografts [144]. Through HO-1 downregulation, FMD reverses chemotherapy-induced immunosuppression by increasing CD8^+^ tumor-infiltrating lymphocyte-dependent cytotoxicity and by reducing tumor-associated Tregs [144].

#### 1.4.2. Human Studies

**Figure 2:**
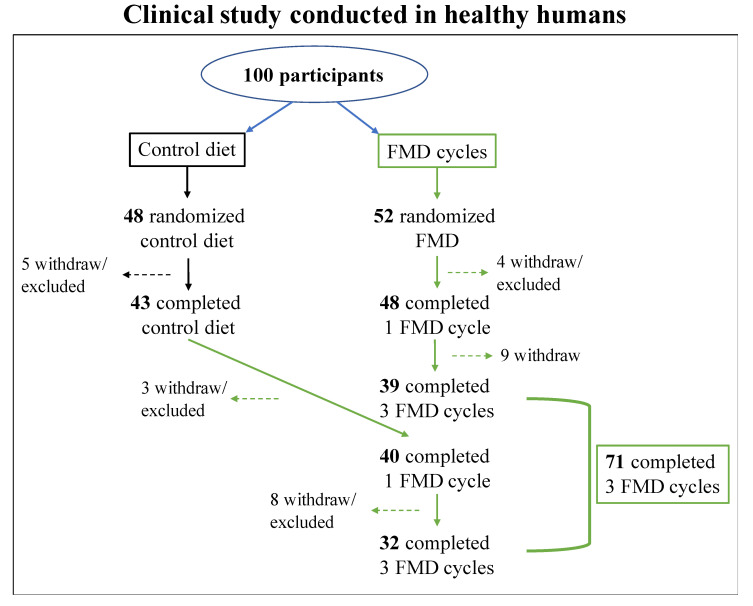
CONSORT diagram. CONSORT diagram of participants selected for the clinical study described by Brandhorst et al., and Wei et al. [112,140]. In the clinical study, 100 participants were enrolled; 48 subjects were randomized to a control group and were asked to continue their normal diet for 3 months and then were subjected to 3 FMD cycles (16 participants withdrew or were excluded throughout the study). Instead, 52 subjects were randomized to the FMD group; FMD was provided for 5 consecutive days per month, for 3 months (13 subjects withdrew or were excluded throughout the study). [112,140].

To investigate the feasibility and impact of FMD in humans, clinical trials were conducted in healthy humans (Figure 2) [112,140]. Participants were subjected to 5 days FMD per month for 3 months in a row and were asked to resume their normal diet after the FMD period. Components and nutrients in the human FMD were selected for their ability to reduce circulating IGF-1 and glucose and to increase IGFBP-1 and the production of ketone bodies, according with the effects of FMD obtained in preclinical studies in rodents. The human FMD diet is composed approximately by at least 11–14% of proteins, 42–43% of carbohydrates and 44–46% of fats and was designed to provide between 34–54% of the normal caloric intake; thus, fat and complex carbohydrates are the higher sources of calories in the FMD regimen [112,140].

Subjects undergoing FMD cycles reported a 11.3% decrease in blood glucose levels and a ~24% reduction in circulating IGF-1, which remained, respectively ~6% and ~15% lower than baseline levels even after the refeeding period. Moreover, participants subjected to FMD showed a 3.7-fold increase in serum ketone bodies and a 1.5-fold increase in IGFBP-1, which returned to baseline levels after resuming the normal diet [112,140]. In addition, FMD decreased systolic blood pressure and reduced bodyweight, waist circumference, total body and trunk fat by 3%, while it increased the relative lean body mass, suggesting that it causes only loss in fat mass [112,140]. Studies conducted in humans report that three FMD cycles reduce the serum level of C-reactive protein (CRP), a marker of inflammation, in subjects classified as at-risk for age-related pathologies than in those subjects who had risk factor values within the normal range, suggesting that FMD also promotes anti-inflammatory effects [112,140]. In general, FMD cycles were well tolerated and no serious adverse events (according to Common Terminology Criteria for Adverse Events) were reported.

**Figure 3:**
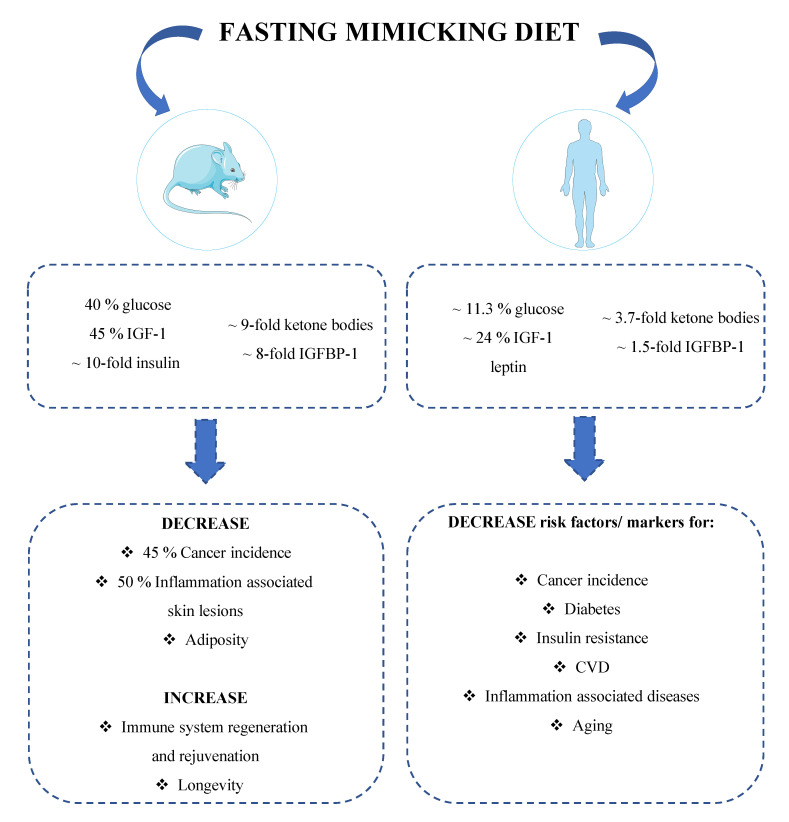
A periodic diet that mimics fasting promotes longevity and reduces risk factors involved in age-related diseases. The fasting mimicking diet (FMD) is characterized by low proteins and sugars and relatively high unsaturated fats. This dietary regimen is able to mimic the metabolic changes induced by water-only fasting while lowering the burden and risk for side effects [112,140]. Preclinical studies show that periodic cycles of FMD can significantly reduce blood glucose, IGF-1, leptin and insulin levels in mice, while increasing ketone bodies production and IGFBP-1 levels. These metabolic changes promote immune system regeneration and rejuvenation while reducing neoplasia incidence by 45%, inflammation associated skin lesions by 50% and obesity [112]. Analogously to rodents, also in humans, periodic FMD decreases blood glucose, insulin, IGF-1, and leptin levels while increasing ketone bodies and IGFBP-1, metabolic changes partially maintained even after the refeeding period [112,140]. These FMD-dependent effects are of particular interest given their key role in age-related diseases, especially cancer and metabolic disorders. Taken together, these data indicate that periodic cycles of FMD could prevent obesity and reduce cancer risk in humans, as suggested by results obtained in preclinical trials [112].

The FMD-dependent reduction in blood glucose and IGF-1 levels is of interest given their key role in age-related diseases, including cancer [6,10,22,68,145]. Collectively, these data indicate that periodic use of cyclic FMD could potentially prevent obesity- or inflammation-related diseases and also reduce cancer risk in humans, as it was shown in rodents [112] (Figure 3).

### 1.5. Alternative Interventions to Reduce Age-Related Diseases Risk Factors

Aging is frequently associated with impaired glucose tolerance and hyperinsulinemia, due to an increase in insulin secretion as a result of high glucose levels [146]. A decline in glucose tolerance is often associated with an increased risk of developing atherosclerosis or non-insulin dependent diabetes mellitus (NIDDM) [147,148]. 

Endurance exercise training reduces insulin levels both during fasting and feeding periods. Several studies showed that individuals who practice exercise periodically have improved glucose tolerance and responsiveness to insulin [149,150,151,152,153]. Seals et al., showed that regular exercise prevented the decline in glucose tolerance and hyperinsulinemia development in older people [152]. Moreover, exercise training was reported to normalize glucose tolerance by reducing insulin resistance in patients with mild NIDDM or impaired glucose tolerance (IGT) [154]. Furthermore, consuming a low-calorie and low-protein vegan diet, composed of unprocessed and uncooked plant-derived foods, for at least two years, or performing endurance exercise are associated with a decrease in cardiometabolic risk [155]. In particular, they reduced the plasma concentrations of lipids, lipoproteins, glucose, insulin, C-reactive protein (CRP) and systolic and diastolic blood pressure [155].

In addition, a meta-analysis study showed that physical activity is associated with lower risk of 13 different kinds of cancer, while another study showed that exercise can reduce the risk of cancer recurrence or progression in certain solid tumors [156,157]. However, additional studies are required to deepen the understanding of the association between exercise and cancer prevention. 

Metformin is another drug that has been reported to attenuate the progression of aging. Metformin is mostly used for the treatment of type 2 diabetes since it decreases glucose production by the liver and increases insulin sensitivity. Several studies showed that metformin can inhibit several nutrient sensing systems, including the somatotropic axis GH/IGF-1, the mTOR signaling and AMPK [158,159,160,161,162,163,164,165,166]. Moreover, it can also lower oxidative stress via mitochondrial complex 1 inhibition and it is reported to reduce DNA damage by decreasing ROS levels and activating DNA damage response mechanisms [167,168,169]. Thus, metformin is likely to be effective against aging in part by affecting the same genes and pathways that mediate the effects of fasting on longevity and disease.

## 2. Conclusions

Studies in simple organisms and mice, demonstrate the link between nutrients and particularly protein intake, growth factors, DNA damage and cancer. The effect of growth factors on DNA damage and cancer is mediated, at least in part, by oxidative stress and damage, but in part also by the inhibition of apoptosis. The reduced activity of growth factors and the lowering of oxidation and DNA damage not only decreases cancer but also extends longevity, since aging is the most important factor promoting cancer. Calorie restriction is a powerful anti-aging intervention, but it also forces the organism into an extremely low nourishment state, which may not constitute malnourishment in the short-term but which may do so long-term. Interventions such as IF and PF are emerging as alternatives to CR, with some of them being able to minimize side effects and burden while maximizing efficacy. Studies on PF have also pointed to 2 key processes absent or low in CR and IF: (a) a pronounced breakdown process both at the intracellular (autophagy etc) and cellular (apoptosis) levels requiring 2 or more days and associated with a high ketogenic state, (b) a rebuilding/regeneration process involving stem and progenitor cells in multiple system and associated with the return from PF to normal feeding (re-feeding). The FMD developed and studied by our laboratories is emerging as a viable and effective intervention in the longevity and cancer prevention fields, since it does not require chronic treatment, it does not cause malnourishment or loss of muscle mass and may be effective when performed only a few times a year for 5 days. In the future years it will be important to continue to test different nutritional interventions with the potential to extend the health span and prevent cancer, with a focus on those that are safe and feasible for long-term use in humans.

## Figures and Tables

**Table 1:** Metabolic, molecular and cellular mechanisms induced by CR to prevent cancer.

**Calorie Restriction** **(CR)**	**Metabolic Adaptations**	**Molecular Adaptations**	**Cellular Adaptations**
	↓ IGF-1 ↓ Insulin ↓ Oxidative stress ↓ Inflammation ↑ Cortisol	↓ PI3K/Akt/S6K ↓ mTOR ↓ Ras/MAPK ↑ Nrf2 ↑ FOXO ↑ PTEN	↓ Cell proliferation ↓ Oxidative damage ↑ DNA repair ↑ Genome instability

**Table 2:** Dietary approaches to promote health span.

	Type of Fasting	Schedule	Description
**Intermittent Fasting** **(IF)**	ADF	24 h fast/ 24 h eating period	Water only fasting every other day
	5:2	2 days fast or very low calorie consumption (500–700 kcal)/ 5 days eating period	Alternation of 2 days of very low-calorie consumption with a 5 days ad libitum re-feeding period
	TRF	12- to 18 h fast/ 6- to 12 h eating period	Food intake resctricted to 6–12 h per day
**Periodic Fasting** **(PF)**	Prolonged fasting	2–5 days of water fast/ 7 days eating period (or longer)	Water only fasting period followed by an ad libitum re-feeding period
	Prolonged FMD	4- to 7 days FMD/ 10- to 25 days eating period	30–50% of the normal caloric intake using a fasting mimicking diet for 4–7 days followed by an ad libitum re-feeding period
